# Effects of Mobile Health Prompts on Self-Monitoring and Exercise Behaviors Following a Diabetes Prevention Program: Secondary Analysis From a Randomized Controlled Trial

**DOI:** 10.2196/12956

**Published:** 2019-09-05

**Authors:** Megan M MacPherson, Kohle J Merry, Sean R Locke, Mary E Jung

**Affiliations:** 1 School of Health and Exercise Sciences University of British Columbia Kelowna, BC Canada; 2 Mechatronic Systems Engineering School of Engineering Science Simon Fraser University Surrey, BC Canada

**Keywords:** self-monitoring, health behavior, prompts, mHealth, mobile apps, exercise, high-intensity interval training, reminder system

## Abstract

**Background:**

A number of mobile health (mHealth) apps exist that focus specifically on promoting exercise behavior. To increase user engagement, prompts, such as text messages, emails, or push notifications, are often used. To date, little research has been done to understand whether, and for how long, these prompts influence exercise behavior.

**Objective:**

This study aimed to assess the impact of prompts on mHealth self-monitoring and self-reported exercise in the days following a prompt and whether these effects differ based on exercise modality.

**Methods:**

Of the possible 99 adults at risk for developing type II diabetes who participated in a diabetes prevention program, 69 were included in this secondary analysis. Participants were randomly assigned to 1 of the following 2 exercise conditions: high-intensity interval training or moderate-intensity continuous training. In the year following a brief, community-based diabetes prevention program involving counseling and supervised exercise sessions, all participants self-monitored their daily exercise behaviors on an mHealth app in which they were sent personalized prompts at varying frequencies. mHealth self-monitoring and self-reported exercise data from the app were averaged over 1, 3, 5, and 7 days preceding and following a prompt and subsequently compared using *t* tests.

**Results:**

In the year following the diabetes prevention program, self-monitoring (t_68_=6.82; *P*<.001; d=0.46) and self-reported exercise (t_68_=2.16; *P*=.03; d=0.38) significantly increased in the 3 days following a prompt compared with the 3 days preceding. Prompts were most effective in the first half of the year, and there were no differences in self-monitoring or self-reported exercise behaviors between exercise modalities (*P* values >.05). In the first half of the year, self-monitoring was significant in the 3 days following a prompt (t_68_=8.61; *P*<.001; d=0.60), and self-reported exercise was significant in the 3 days (t_68_=3.7; *P*<.001; d=0.37), 5 days (t_67_=2.15; *P*=.04; d=0.14), and 7 days (t_68_=2.46; *P*=.02; d=0.15) following a prompt, whereas no significant changes were found in the second half of the year.

**Conclusions:**

This study provides preliminary evidence regarding the potential influence of prompts on mHealth self-monitoring and self-reported exercise and the duration for which prompts may be effective as exercise behavior change tools. Future studies should determine the optimal prompting frequency for influencing self-reported exercise behaviors. Optimizing prompt frequency can potentially reduce intervention costs and promote user engagement. Furthermore, it can encourage consumers to self-monitor using mHealth technology while ensuring prompts are sent when necessary and effective.

**International Registered Report Identifier (IRRID):**

DERR2-10.2196/11226

## Introduction

### Background

Mobile phones are ubiquitous and becoming an integral part of daily life. In 2015, global subscriptions of mobile phones were approximately 7 billion; this constitutes a substantial increase from 738 million subscriptions in 2000 [1]. In addition, 95% of the global population resides in areas covered by cellular networks, the majority of which has the opportunity to access the internet through their mobile devices, as mobile broadband networks (3G or above) reach approximately 84% of the global population [2]. Smartphones are internet-enabled mobile phones that possess a multitude of capabilities through the use of electronic apps, which are specifically developed to be used on a handheld device for various purposes. In fact, a survey by Bender et al [3] examining mobile phone usage among white, Filipinos, Koreans, and Latino Americans found that individuals are more likely to access the internet through mobile phones when compared with computers, and that mobile phone usage did not significantly differ between these groups.

As the widespread adoption of mobile phones increases, so too does the opportunity for the development and implementation of theory-driven, cost-effective, evidence-based mobile phone apps (ie, mobile health [mHealth] app) used to influence health behaviors. An mHealth app is any mobile phone app, which is used for tracking, guiding, teaching, or enabling individuals in any health-related behaviors and can range from tracking diet and exercise to guided meditation or monitoring of diabetic sugar level. The accessibility of these apps is also advantageous for researchers who can monitor consumer behaviors remotely, provide real-time feedback, and aggregate data so as to improve monitoring systems [4]. Despite the rapidly growing number of mHealth apps on the market and the advantages they may afford to consumers and researchers alike, there is a profound lack of theory-driven, evidence-based mHealth apps [5-8].

### Mobile Health and Behavior Change Techniques

This lack of evidence-based mHealth apps may be because of the time-consuming nature of conventional methods of evaluation, such as randomized controlled trials (RCTs), being unable to keep up with the dynamic nature of mHealth app development, and the rapid advancement of mobile technologies [9]. One approach to address this issue has been to research the irreducible, replicable, and observable components—known as behavior change techniques (BCTs)—of mHealth interventions [10,11]. BCTs most frequently used within physical activity (PA) mHealth apps include self-monitoring of behavior, feedback on behavior, and prompts or cues [12,13].

Self-monitoring is a commonly used and robust BCT, which often involves participants logging target behaviors [14]. A meta-regression by Michie et al [15] found that interventions, which included self-monitoring, were more effective at improving PA than those that did not. Within mHealth literature, self-monitoring has been shown to improve PA and dietary behaviors [16,17]. Carels et al [18] posit that daily self-monitoring may allow individuals to increase their awareness of the target behavior, thus allowing them to implement strategies to resume a behavior when they become aware that they are not engaging in the target behavior. In support of this, studies have shown that adherence to daily self-monitoring is associated with increased weight loss [19], and self-monitoring of daily exercise is associated with increased PA and weight loss [18]. Furthermore, research has suggested that self-monitoring and adherence to PA goals may be bolstered through the use of personalized prompts or feedback [20]. Specifically, 1 study found that individuals who received personalized goal setting prompts logged significantly more PA than their counterparts who received generic prompts [21].

Prompts within mHealth apps promote individual-app interaction (eg, text messages, multimedia message services, and mobile phone push notifications). There is a growing body of evidence to support the use of prompts as either stand-alone interventions or supplementary features to increase the effectiveness of health interventions [22,23]. Specifically, reviews have shown that prompts may be effective in enhancing diet or weight loss, PA behaviors, and smoking cessation behaviors [24-27]. That said, few interventions parse out and examine the influence of prompts. Prompt interventions targeting health behaviors are often short in duration, lasting less than 14 weeks on average [22,28,29], and vary in the frequency of prompts delivered from daily to weekly or monthly messages [26,30]. This variability in design, coupled with the fact that few studies have reported on or assessed the effectiveness of individual intervention characteristics [26,31], demonstrates that informative research is required to understand ideal message frequency targeting behavior modification.

### Purpose

This paper analyzes mobile phone prompt data to promote exercise adherence for 1 year following a diabetes prevention program research study. Program participants were randomized to perform 1 of the following 2 exercise modalities: high-intensity interval training (HIIT) or moderate-intensity continuous training (MICT). HIIT has garnered attention as an exercise program primarily because of its shorter duration and similar cardiometabolic health effects when compared with MICT [32,33]. There may be a differential impact of prompts on cuing the engagement of time-efficient HIIT compared with MICT. Previous studies highlighting the positive impact prompts have on promoting PA have primarily used MICT to examine outcomes such as walking behaviors, daily step count, and sedentary behaviors [22] but have yet to examine the impact on HIIT engagement.

The main objective of this study was to examine whether mHealth prompts influence self-monitoring and self-reported exercise in 1, 3, 5, or 7 days following a prompt. Prompts are meant to provide brief effects; therefore, we hypothesized that there would be initial increases in both mHealth self-monitoring and self-reported exercise behaviors. No specific hypotheses on whether prompt effects would last 1, 3, 5, or 7 days were made. Given the short follow-up durations of previous research and lack of literature addressing the impact of prompts on exercise prescriptions, we wanted to explore whether the effects of a prompt were consistent in the first and second half of the year following a diabetes prevention program and whether the impact of prompts differed between those randomized to HIIT or MICT.

## Methods

### Overview

This paper presents a secondary analysis examining the effect of personalized mHealth prompts on self-monitoring and self-reported exercise behaviors within a diabetes prevention program. Complete details regarding the study design, methods, and procedures have been previously published [34]. The program was a 2-week lifestyle modification program aimed at reducing type II diabetes risk (ClinicalTrials.gov; NCT02164474). This program consisted of 7 one-on-one sessions with a trained exercise counselor focusing on brief counseling, self-regulatory skills development, and exercise. A total of 99 individuals (69 of which were included in this secondary analysis) participated in a diabetes prevention program and were randomly assigned to 1 of 2 exercise conditions: HIIT or MICT. HIIT involves alternating bursts of vigorous-intensity exercise with a recovery period of light exercise, whereas MICT encompasses exercising at a steady pace for a longer duration. Following the program completion, all participants were prescribed 3 days of exercise and permitted up to 4 rest days per week to be used at the discretion of the participant. Participants in the MICT group were prescribed 150 min of weekly moderate-intensity exercise (50 min 3 times per week), whereas HIIT participants were prescribed 75 min of vigorous interval exercise (25 min of intervals 3 times per week). To promote exercise adherence in free-living conditions 1 year following the diabetes prevention program, participants were provided with an mHealth app (or paper logbook if they opted not to use the app) to encourage exercise self-monitoring for 1 year immediately following completion of the intervention.

#### Mobile Health App

The theory-based mHealth app used in the diabetes prevention program was designed using principles from social cognitive theory to help participants self-monitor their exercise behaviors [35]. Participants were encouraged to self-monitor their exercise behaviors (including days in which they did not exercise) through the mHealth app. The app was designed to allow each participant to self-monitor their daily exercise behaviors; possible responses included “yes I exercised today,” “rest day,” and “no I did not exercise today.” If a participant responded with “yes I exercised today,” they were asked additional questions regarding the type, duration, and intensity of their exercise session. Participants were rewarded with points on the app for continual self-monitoring and exercise engagement. Feasibility testing of self-monitoring through this app has demonstrated increased self-monitoring and PA behaviors over an 8-week period for those who used the app when compared with a control group [35].

The messaging platform within this mHealth app allowed for 2-way messaging between participants and their exercise counselors. Participants received personalized messages that encompassed counselors sending name-specific prompts using a series of message templates (Table 1). These messages were based on social cognitive theory and modeled off of those used by Voth et al, which targeted self-monitoring, verbal persuasion, and performance accomplishment [35]. Exercise counselors sent their participants 1 message per month and would respond to participants’ messages with social or instrumental support to reinforce the aforementioned behavior change concepts. Participants were also sent a reminder message to self-monitor if they failed to self-monitor for 3 consecutive days. A *prompt* was defined as any of the above message types in which there was a minimum of 6 days preceding it with no other message. This definition was determined to exclude any subsequent conversation resulting from a prompt in the analyses.

**Table 1:** Example messages to participants.

Message type	Example message
Reminder to self-monitor	Hi (insert name), I noticed that you have not checked in with your exercise for the past few days. Is everything ok? Let me know how I can help you. You can do this!
Use of verbal persuasion and self-set rewards	Hi (insert name)! Just dropping a note to say how proud I am of all of your hard work over these past few months. Wow - you've been working hard towards being a regular exerciser for over half a year! Very impressive. Few things in life come easy - motivating yourself to exercise consistently is no exception. We are so proud of you for each and every exercise bout you do - because we know firsthand how difficult it is. So keep up the good work! And while you're at it - start acknowledging all of your hard work and REWARD yourself! A bath, a glass of wine, 10 minutes of peace and quiet, whatever it is - give it yourself after you complete your next exercise bout. You deserve it.
Importance of self-monitoring	Wow – how time flies when you’re doing fabulously! (insert name) – really impressed with your exercise behaviour, but equally impressed by your faithful check-ins. Keeping tabs on what you’re doing keeps you honest, so make sure you continue to self-monitor here. And remember, self-monitoring is most important when you miss a day – so report that if it happens! You’re human!
Performance accomplishment	Hey (insert name). I have been watching your progress for the last few weeks and wanted to say congratulations on what an awesome job you have been doing! You should be really proud of yourself – you’ve been sticking with your exercise plan over the past month! Keep up this fantastic effort and I'll be right here watching your fabulous achievements.
Response to participant (providing social support)	I love your attitude (insert name), and your perseverance! I'm glad you can recognize the changes you have achieved, but also strive for more. Keep pushing through and you will get there!
Response to participant (providing instrumental support)	Hi (insert name), we are having some trouble with the system. I have unlocked yesterday for you so hopefully you can re-enter your exercise and it works! Let me know.

### Participants

Of 99 adults participated in a diabetes prevention program, 69 (51 females, 17 males, and 1 missing; mean age 50.7 years, SD 9.4) were included in this analysis. Participants were eligible to participate if they were between the ages of 30 and 65 years, were inactive (defined as engaging in <3 bouts of moderate or vigorous aerobic exercise per week in the past 6 months), had a body mass index between 24 and 40 kg/m^2^, and were cleared to engage in vigorous exercise using Canadian Society for Exercise Physiology Physical Activity Readiness Questionnaire-Plus [36]. Participants were asked to provide demographic information including age, ethnicity, highest level of education completed, and current occupational status. Only individuals who chose to self-monitor through the mHealth app were included in this analysis; an additional 30 participants were not included because of the use of paper logbook (n=7), using the app for less than 2 months throughout the 1-year follow-up period (n=14; self-monitored an average of 40 days), and data error (n=9).

### Outcome Measures

Outcome measures include frequency of both mHealth self-monitoring and self-reported exercise in the week before and after a prompt. mHealth self-monitoring was defined as any day in which a participant logged on the mHealth app; this includes days in which they engaged in purposeful exercise, rest days, and days in which they did not exercise and exceeded their number of rest days. Self-reported exercise was defined as only those days in which participants logged on the mHealth app that they engaged in purposeful exercise. Specifically, when a participant self-monitored “yes I exercised today,” they were able to type in the details of their exercise; however, for the purpose of this study, the level or type of logged exercise was not examined.

### Procedures

During the one-on-one counseling sessions, the exercise counselor created each participant’s profile on the mHealth app and taught participants how to self-monitor their exercise to ensure participants were confident in their ability to monitor their exercise *.* Throughout the free-living 1-year follow-up period, participants were sent personalized messages delivered through the app messaging system from their exercise counselor at a variable frequency. A *prompt* was defined as any message in which there was a minimum of 6 days preceding it with no other message. This means that any subsequent conversation resulting from a prompt was not included in the analyses.

#### Data Acquisition

The following procedures were completed using MATLAB (MathWorks, Inc) in to extract outcome measures from app data regarding daily activity; these measures include mean mHealth self-monitoring and self-reported exercise in the week before and after a prompt. Participants’ daily activity on the mHealth app was initially coded as (1) logged “yes I exercised today,” (2) logged “no I did not exercise today,” (3) logged “rest day,” and (4) did not log anything. Following this, participants logging was dichotomously categorized: *mHealth self-monitoring* (1-3=yes and 4=no) and *self-reported exercise* (1=yes and 2-4= no).

To determine if self-monitoring behaviors increased in the days following a prompt, the average number of days self-monitored in 1, 3, 5, and 7 days preceding and following a prompt was calculated. These days were selected to facilitate analysis on how the brief effects of a prompt on self-monitoring and self-reported exercise may vary over the week. Days 2, 4, and 6 were excluded to decrease the number of *t* tests being run in an attempt to decrease type I error. Once averages for individual prompts were calculated, weekly averages were established for the whole year, months 1 to 6, and months 7 to 12; these time points are in line with the overall research study, which assessed all main outcomes at 6 and 12 months. The same procedures were followed to identify the average number of days for self-reported exercise.

### Analysis

Paired samples *t* tests were conducted to determine whether self-monitoring and self-reported exercise differed (1) in the day following a prompt compared with the day preceding a prompt, (2) in the 3 days following a prompt compared with the 3 days preceding a prompt, (3) in the 5 days following a prompt compared with the 5 days preceding a prompt, and (4) in the 7 days following a prompt compared with the 7 days preceding it. This analytic procedure was chosen as it aligns with the nature of our hypotheses examining differences before and after a prompt. Change scores for mHealth self-monitoring and self-reported exercise were calculated by taking the difference between the days before and after a prompt. Independent samples *t* tests were conducted to compare change scores between those randomized to HIIT and MICT. Analyses were completed independently for the whole year (months 1-12); the first half of the year (months 1-6) and the latter half of the year (months 7-12). All data were analyzed using SPSS statistics for Windows (version 21, SPSS Inc). Significance level was set at *P*<.05. Effect sizes were calculated using Cohen *d*.

## Results

### Months 1 to 12

Baseline measurements and demographics of the 69 inactive and overweight adults (mean age 50.7 years, SD 9.40) whose data were included in this study are reported in Table 2. A total of 32 participants were randomized to HIIT, and 37 were randomized to MICT. During the free-living 1-year follow-up period, a total of 369 prompts were sent to the HIIT group (mean 10.25 per participant, SD 3.05), and 465 prompts were sent to the MICT group (mean 10.11 per participant, SD 4.29).

In the year following a diabetes prevention program, there were no significant increases in mHealth self-monitoring or self-reported exercise in 1, 5, and 7 days following a prompt compared with the days preceding a prompt. Both mHealth self-monitoring and self-reported exercise did significantly increase in the 3 days following a prompt compared with the 3 days preceding it.

There were no significant differences between exercise conditions (HIIT and MICT) for both mHealth self-monitoring and self-reported exercise in 1, 3, 5, and 7 days following a prompt. Descriptive statistics and inferential statistics are given in Tables 3 and 4, respectively.

**Table 2:** Descriptive statistics for individuals who took part in the intervention.

Characteristics		All (N=69)	HIIT^a^ (n=32)	MICT^b^ (n=37)
Age (years), mean (SD)		50.70 (9.40)	50.72 (9.01)	50.61 (9.87)
**Gender, n (%)**				
	Male	17 (25)	5 (16)	12 (32)
	Female	51 (74)	26 (81)	25 (68)
	Did not answer	1 (1)	1 (3)	0 (0)
Body mass (kg), mean (SD)		87.92 (19.87)	87.54 (22.35)	88.26 (17.70)
Waist circumference (cm), mean (SD)		107.02 (14.31)	106.66 (14.80)	107.34 (14.05)
VO_2_ relative (mL/kg/min)^c^, mean (SD)		22.77 (5.88)	22.23 (4.92)	23.20 (6.64)
**Ethnic origin, n (%)**				
	Caucasian	60 (87)	27 (85)	33 (85)
	Latin American	2 (3)	1 (3)	1 (3)
	Asian	2 (3)	1 (3)	1 (3)
	Aboriginal	1 (1)	1 (3)	1 (3)
	Other	2 (3)	1 (3)	1 (3)
	Missing	2 (3)	1 (3)	1 (3)
**Annual income (Can $), n (%)**				
	0-24,999	1 (2)	0 (0)	1 (3)
	25,000-49,999	5 (7)	2 (6)	3 (8)
	50,000-74,999	14 (20)	8 (25)	6 (16)
	75,000-99,999	14 (20)	9 (28)	5 (13)
	>100,000	33 (48)	12 (38)	21 (57)
	Missing	2 (3)	1 (3)	1 (3)
**Education, n (%)**				
	High school	9 (13)	5 (15)	4 (11)
	College diploma	22 (32)	13 (41)	9 (24)
	Bachelor’s degree	20 (29)	6 (19)	14 (38)
	Postgraduate degree	15 (22)	6 (19)	9 (24)
	Missing	3 (4)	2 (6)	1 (3)
**Marital status, n** **(%)**				
	Single	6 (9)	3 (10)	3 (8)
	Married	49 (72)	25 (78)	24 (65)
	Common law	5 (7)	1 (3)	4 (11)
	Divorced	5 (7)	1 (3)	4 (11)
	Widowed	1 (1)	1 (3)	0 (0)
	Missing	3 (4)	1 (3)	2 (5)

^a^HIIT: high-intensity interval training.

^b^MICT: moderate-intensity continuous training.

^c^Cardiorespiratory fitness was the primary outcome of the diabetes prevention program. Participants completed a maximal cardiorespiratory fitness (VO_2peak_) test to exhaustion on a cycle ergometer at baseline and 6- and 12-month follow-ups.

**Table 3:** Average number of days participants self-monitored and self-reported exercise in a mobile health app in 1, 3, 5, and 7 days before and after a prompt in months 1 to 12.

Days^a^		Total, mean (SD)		HIIT^b^, mean (SD)		MICT^c^, mean (SD)	
		Before	After	Before	After	Before	After
**1**							
	SM^d^	0.86 (0.15)	0.87 (0.16)	0.88 (0.11)	0.86 (0.14)	0.85 (0.17)	0.87 (0.17)
	Exercise	0.44 (0.20)	0.45 (21)	0.40 (0.16)	0.42 (0.21)	0.48 (0.23)	0.49 (0.21)
**3**							
	SM	2.41 (0.42)	2.60 (0.41)	2.44 (0.34)	2.59 (0.36)	2.38 (0.48)	2.61 (0.46)
	Exercise	1.21 (0.48)	1.40 (0.53)	1.16 (0.38)	1.27 (0.45)	1.44 (0.53)	1.51 (0.57)
**5**							
	SM	4.23 (0.73)	4.25 (0.74)	4.27 (0.61)	4.29 (0.59)	4.20 (0.83)	4.21 (0.85)
	Exercise	2.22 (0.79)	2.26 (0.85)	1.94 (0.64)	2.03 (0.73)	2.46 (0.83)	2.46 (0.91)
**7**							
	SM	5.94 (0.99)	5.99 (1.00)	5.99 (0.82)	6.03 (0.81)	5.90 (1.13)	5.96 (1.16)
	Exercise	3.12 (1.09)	3.16 (1.19)	2.75 (0.89)	2.82 (0.96)	3.42 (1.15)	3.46 (1.30)

^a^Comparisons were made between 1 day before and after a prompt, 3 days before and after a prompt, 5 days before and after a prompt, and 7 days before and after a prompt for mHealth self-monitoring and self-reported exercise.

^b^HIIT: high-intensity interval training.

^c^MICT: moderate-intensity continuous training.

^d^SM: mHealth self-monitoring.

**Table 4:** *T* test, *P* values, and effect size of self-monitoring and self-reported exercise in a mobile health app in 1, 3, 5, and 7 days before and after a prompt in months 1 to 12.

Days^a^		Total			HIIT^b^ versus MICT^c^		
		*t* test (df)	*P* value	Cohen *d*	*t* test (df)	*P* value	Cohen *d*
**1**							
^ ^	SM^d^	0.16 (68)	.87	0.06	1.87 (67)	.07	0.46
	Exercise	0.73 (68)	.47	0.05	0.09 (67)	.93	0.02
**3**							
	SM	6.82 (68)	<.001	0.46	1.25 (67)	.22	0.31
	Exercise	2.16 (68)	.03	0.38	0.46 (67)	.65	0.11
**5**							
	SM	0.46 (68)	.65	0.03	0.05 (67)	.97	0.01
	Exercise	0.89 (68)	.38	0.05	1.03 (67)	.31	0.25
**7**							
	SM	1.18 (68)	.24	0.09	0.17 (67)	.86	0.04
	Exercise	0.99 (68)	.33	0.04	0.18 (67)	.86	0.04

^a^Comparisons were made between 1 day before and after a prompt, 3 days before and after a prompt, 5 days before and after a prompt, and 7 days before and after a prompt for mHealth self-monitoring and self-reported exercise.

^b^HIIT: high-intensity interval training.

^c^MICT: moderate-intensity continuous training.

^d^SM: mHealth self-monitoring.

### Months 1 to 6

In months 1 to 6, a total of 226 prompts were sent to the HIIT group (mean 6.28 per participant, SD 1.77), and 283 prompts were sent to the MICT group (mean 6.15 per participant, SD 1.85).

In the first 6 months following the program, mHealth self-monitoring significantly increased in the 3 days following a prompt compared with the 3 days preceding it but did not significantly differ in 1, 5, or 7 days following a prompt compared with preceding days. Self-reported exercise did not significantly increase in the day following a prompt compared with the day preceding it; however, it did significantly increase in 3, 5, and 7 days following a prompt compared with the respective preceding days.

In 1, 5, and 7 days following a prompt compared with the days preceding it, there were no significant differences between HIIT and MICT groups for both mHealth self-monitoring and self-reported exercise. In the 3 days following a prompt compared with the 3 days preceding it, independent samples *t* tests conducted on change scores suggest that there was a significantly larger change in self-monitoring for those randomized to MICT compared with those randomized to HIIT (*t*_67_=2.2; *P*=.03; *d*=0.54), but no significant group differences for self-reported exercise (*t*_67_=0.05; *P*=.96; *d*=0.012). When looking at HIIT and MICT independently, both groups demonstrated significant increases between the 3 days before and after a prompt in mHealth self-monitoring (HIIT: *t*_31_=4.44; *P*<.001; *d*=0.64; MICT: *t*_36_=7.94; *P*<.001; *d*=0.90). Additional information regarding descriptive statistics and inferential statistics are given in Tables 5 and 6, respectively.

**Table 5:** Average number of days participants self-monitored and self-reported exercise in a mobile health app in 1, 3, 5, and 7 days before and after a prompt in months 1 to 6.

Days^a^		Total, mean (SD)		HIIT^b^, mean (SD)		MICT^c^, mean (SD)	
		Before	After	Before	After	Before	After
**1**							
	SM^d^	0.90 (0.17)	0.89 (0.18)	0.93 (0.12)	0.89 (0.18)	0.87 (0.20)	0.89 (0.17)
	Exercise	0.44 (0.26)	0.49 (0.26)	0.42 (0.22)	0.47 (0.26)	0.46 (0.29)	0.51 (0.27)
**3**							
	SM	2.40 (0.39)	2.70 (0.37)	2.46 (0.32)	2.68 (0.37)	2.35 (0.44)	2.72 (0.38)
	Exercise	1.31 (0.53)	1.52 (0.59)	1.20 (0.47)	1.41 (0.54)	1.40 (0.57)	1.62 (0.62)
**5**							
	SM	4.41 (0.68)	4.40 (0.75)	4.49 (0.59)	4.45 (0.65)	4.35 (0.75)	4.36 (0.83)
	Exercise	2.32 (0.86)	3.45 (0.98)	2.10 (0.83)	2.24 (0.89)	2.51 (0.86)	2.64 (1.01)
**7**							
	SM	6.21 (0.90)	6.21 (1.00)	6.31 (0.78)	6.28 (0.83)	6.12 (1.00)	6.15 (1.13)
	Exercise	3.26 (1.21)	3.45 (1.27)	2.98 (1.12)	3.11 (1.04)	3.50 (1.24)	3.74 (1.39)

^a^Comparisons were made between 1 day before and after a prompt, 3 days before and after a prompt, 5 days before and after a prompt, and 7 days before and after a prompt for mHealth self-monitoring and self-reported exercise.

^b^HIIT: high-intensity interval training.

^c^MICT: moderate-intensity continuous training.

^d^SM: mHealth self-monitoring.

**Table 6:** *T* test, *P* values, and effect size of self-monitoring and self-reported exercise in a mobile health app in 1, 3, 5, and 7 days before and after a prompt in months 1 to 6.

Days^a^		Total			HIIT^b^ versus MICT^c^		
		*t* test (df)	*P* value	Cohen *d*	*t* test (df)	*P* value	Cohen *d*
**1**							
	SM^d^	0.61 (68)	.05	0.06	1.53 (67)	.13	0.37
	Exercise	1.40 (68)	.17	0.19	0.09 (67)	.93	0.02
**3**							
	SM	8.61 (68)	<.001	0.60	2.20 (67)	.03	0.54
	Exercise	3.70 (68)	<.001	0.37	0.05 (67)	.96	0.01
**5**							
	SM	0.38 (68)	.71	0.01	0.51 (67)	.62	0.12
	Exercise	2.15 (68)	.04	0.14	0.09 (67)	.93	0.02
**7**							
	SM	0.06 (68)	.95	0.01	0.52 (67)	.60	0.13
	Exercise	2.46 (68)	.02	0.15	0.80 (67)	.43	0.20

^a^Comparisons were made between 1 day before and after a prompt, 3 days before and after a prompt, 5 days before and after a prompt, and 7 days before and after a prompt for mHealth self-monitoring and self-reported exercise.

^b^HIIT: high-intensity interval training.

^c^MICT: moderate-intensity continuous training.

^d^SM: mHealth self-monitoring.

### Months 7 to 12

In months 7 to 12, a total of 143 prompts were sent to the HIIT group (mean 4.47 per participant, SD 1.5), and 182 prompts were sent to the MICT group (mean 4.92 per participant, SD 2.41).

In the second half of the year following the program, there were no significant differences in either mHealth self-monitoring or self-reported exercise in 1, 3, 5, and 7 days following a prompt compared with the days preceding a prompt. There were no significant differences between exercise conditions (HIIT and MICT) for both mHealth self-monitoring and self-reported exercise in 1, 3, 5, and 7 days following a prompt in the second half of the year. Descriptive statistics and inferential statistics are presented in Tables 7 and 8, respectively.

**Table 7:** Average number of days participants self-monitored and self-reported exercise in a mobile health app in 1, 3, 5, and 7 days before and after a prompt in months 7 to 12.

Days^a^		Total, mean (SD)		HIIT^b^, mean (SD)		MICT^c^, mean (SD)	
		Before	After	Before	After	Before	After
**1**							
	SM^d^	0.82 (0.23)	0.84 (0.24)	0.84 (0.20)	0.84 (0.23)	0.80 (0.25)	0.84 (0.25)
	Exercise	0.44 (0.28)	0.43 (0.29)	0.38 (0.25)	0.35 (0.29)	0.49 (0.29)	0.50 (0.28)
**3**							
	SM	2.41 (0.66)	2.45 (0.69)	2.40 (0.62)	2.45 (0.61)	2.42 (0.71)	2.46 (0.76)
	Exercise	1.31 (0.71)	1.24 (0.65)	1.11 (0.58)	1.06 (0.60)	1.49 (0.76)	1.39 (0.67)
**5**							
	SM	4.00 (1.09)	4.06 (1.03)	4.02 (1.01)	4.08 (0.96)	3.98 (1.16)	4.04 (1.09)
	Exercise	2.12 (1.02)	2.06 (0.96)	1.76 (0.81)	1.78 (0.84)	2.42 (1.09)	2.32 (1.00)
**7**							
	SM	5.58 (1.50)	5.71 (1.43)	5.57 (1.40)	5.69 (1.38)	5.59 (1.61)	5.72 (1.49)
	Exercise	2.93 (1.42)	2.87 (1.38)	2.46 (1.18)	2.46 (1.23)	3.35 (1.49)	3.22 (1.42)

^a^Comparisons were made between 1 day before and after a prompt, 3 days before and after a prompt, 5 days before and after a prompt, and 7 days before and after a prompt for mHealth self-monitoring and self-reported exercise.

^b^HIIT: high-intensity interval training.

^c^MICT: moderate-intensity continuous training.

^d^SM: mHealth self-monitoring.

**Table 8:** *T* test, *P* values, and effect size of self-monitoring and self-reported exercise in a mobile health app in 1, 3, 5, and 7 days before and after a prompt in months 7 to 12.

Days^a^		Total			HIIT^b^ versus MICT^c^		
		*t* test (df)	*P* value	Cohen *d*	*t* test (df)	*P* value	Cohen *d*
**1**							
	SM^d^	1.06 (68)	.29	0.09	0.81 (67)	.42	0.20
	Exercise	0.25 (68)	.80	0.04	0.56 (67)	.58	0.13
**3**							
	SM	0.96 (68)	.34	0.06	0.20 (67)	.85	0.05
	Exercise	1.22 (68)	.23	0.10	0.43 (67)	.67	0.11
**5**							
	SM	1.05 (68)	.29	0.06	0.08 (67)	.94	0.02
	Exercise	0.62 (68)	.05	0.06	0.72 (67)	.47	0.18
**7**							
	SM	1.57 (68)	.12	0.09	0.07 (67)	.95	0.02
	Exercise	0.72 (68)	.48	0.04	0.74 (67)	.46	0.18

^a^Comparisons were made between the 1 day before and after a prompt, the 3 days before and after a prompt, 5 days before and after a prompt, and 7 days before and after a prompt for mHealth self-monitoring and self-reported exercise.

^b^HIIT: high-intensity interval training.

^c^MICT: moderate-intensity continuous training.

^d^SM: mHealth self-monitoring.

## Discussion

### Principal Findings

The primary objective of this secondary data analysis was to assess changes in mHealth self-monitoring and self-reported exercise in the days preceding and following a prompt. Secondary objectives of this research were to examine whether results differed based on exercise modality (HIIT vs MICT) and the differences between the first and second half of the year following a diabetes prevention program. Overall results suggest that both self-monitoring and self-reported exercise behaviors significantly increase in the 3 days following a prompt when compared with the 3 days preceding it, the greatest changes were observed in the first half of the year, and there were no differences between exercise modality.

#### Months 1 to 12

In the year following a diabetes prevention program, the observed differences in self-monitoring and self-reported exercise behaviors were most potent in the 3 days following a prompt, whereas there were no significant changes in 1, 5, or 7 days following a prompt. Exercise is a complex behavior that requires self-regulation such as scheduling and planning [37]. As such, it might be unrealistic to expect to observe changes in self-reported exercise behavior in a singular day or in the day immediately following a prompt. The changes in behaviors before and after a prompt in the year following a diabetes prevention program may be most effective somewhere between 1 and 3 days, as an individual begins to self-regulate to schedule exercise to get back on track.

#### First and Second Half of the Year

In the first half of the year, self-reported exercise behavior significantly increased in 3, 5, and 7 days following a prompt, but not the day immediately following a prompt, whereas no significant changes were observed in the second half of the year. Reasons why prompts appeared to have no observed change on behaviors in the second half of the year are unknown and should be the focus of future research. In the first half of the year, a total of 226 prompts were sent, whereas in the second half of the year, 143 prompts were sent. Although the difference in frequency of prompts sent between the first and second half of the year could have influenced the observed changes, additional research is required to examine the role of prompt frequency in changing self-monitoring and self-reported exercise behavior.

Similar to self-reported exercise, the impact of prompts on mHealth self-monitoring was only observed in the first half of the year. Specifically, in the first half of the year following a diabetes prevention program, mHealth self-monitoring significantly increased in the 3 days following a prompt compared with the 3 days preceding a prompt, but not in 1, 5, or 7 days following a prompt. Individuals included in this analysis self-monitored an average of 286 days in the 12-month follow-up period. It may be the case that prompts are not needed for individuals who regularly self-monitor. However, it is difficult to discern the impact of a prompt on daily self-monitoring behaviors, given that the majority of participants were self-monitoring on a daily basis, and there was no control group.

#### High-Intensity Interval Training Versus Moderate-Intensity Continuous Training

There is a growing body of evidence suggesting HIIT may be a viable exercise alternative to MICT [32,33]. There were no differences in behaviors preceding and following a prompt between the 2 exercise modalities. This may suggest that certain self-regulatory skills and cognitions may not appreciably differ between HIIT and MICT. Although there are compelling arguments for HIIT being a more time efficient and easier to self-manage alternative to MICT [38-42], our results suggest that the impact of prompts on self-monitoring and self-report exercise did not differ between HIIT and MICT.

### Strengths and Limitations

Systematic reviews of the literature have shown that individuals who received prompts had greater weight loss and increased PA compared with nonprompt controls [26,31]. Despite the overall positive effects of prompts, design of these studies has varied significantly, and few studies have reported on or assessed the effectiveness of individual intervention characteristics [26,31]. Within these reviews of the literature, it has been recommended that future research focus on the impact of specific prompt delivery characteristics such as prompt frequency, timing, and intervention duration. A primary strength of this analysis is that it looked at the immediate effect of a prompt on self-monitoring and self-reported exercise behaviors. Another strength of this study was that we examined participants randomly assigned to different exercise conditions (HIIT and MICT), which allowed us to examine differences in prompt effectiveness between exercise modalities, which has not been addressed in previous studies. Finally, the 12-month follow-up period in which participants self-monitored on an mHealth app is longer than previous prompt studies, which often last less than 14 weeks [22,28,29]. Although examining 12 months of self-monitoring was a strength, people may not need to self-monitor in this way. Once people establish a regular behavioral pattern of exercise, self-monitoring through an app may not be needed to facilitate this regular exercise engagement.

Despite these strengths, this study represents a secondary analysis of prompt data, and the primary objectives of this RCT did not relate to mHealth prompts. Limitations of this paper include a lack of a control condition (ie, not receiving prompts), no a priori sample size calculation, and conducting multiple *t* tests without adjustment. All participants using the mHealth app received prompts, and there was no experimental manipulation of prompts. Another limitation was that there had been no validation of this mHealth app as an exercise measure. However, the information participants report on the mHealth app is similar to the information contained in validated measures (eg, Godin-Shephard Leisure-Time Physical Activity Questionnaire [43]). Although we recognize this measure has not been validated, our research question and outcomes concern engagement or nonengagement in exercise. As such, we are less concerned with the validation impacting results as we are simply looking whether or not individuals logged exercise.

One final limitation regarding the criteria for prompts to be included in the analyses. Our analyses examined the effects of a prompt on self-monitoring and self-reported exercise behaviors and did not include any subsequent conversation resulting from a prompt in the analysis. There is a possibility that the amount of interaction between a participant and their exercise counselor on the mHealth app influenced their behaviors.

Despite this preliminary evidence that prompts may influence self-monitoring and self-reported exercise behaviors, future research is needed to examine the causal impact of prompt frequency on self-monitoring and self-reported exercise behavior in an attempt to elucidate an optimal prompt frequency for behavior change.

### Future Directions

These analyses used only those participants who were engaging with the app and individuals self-monitored approximately 80% of the time. In addition, we were unable to analyze whether the level of virtual interaction between exercise counselors and participants influenced the effect of a prompt on self-monitoring and self-reported exercise behaviors. Future studies should address the impact of prompts on less consistent self-monitors while also examining the role that social interaction may play on self-monitoring and self-reported exercise.

The duration of prompts’ impact on self-reported exercise behaviors was relatively short (in 3, 5, and 7 days following a prompt, only in the first half of the year). Future studies should examine the optimal prompt frequency and timing for cueing self-reported exercise behavior following behavior change programs. Utilization of optimization trials or n-of-1 trials may be 1 possible means to examine dose-response relationship between app-delivered prompts and exercise.

### Conclusions

Within this analysis, we provided evidence regarding the observed changes in self-monitoring and self-reported exercise behavior following a prompt and the duration for which prompts may be effective as exercise behavior change tools. Future studies assessing prompts should examine causal factors relating to the observed decrease in prompt effectiveness on self-reported exercise behaviors in the 7 to 12 months following an exercise behavior change program. Understanding how to optimally intervene through prompts can decrease intervention cost and time as researchers may limit unnecessary prompts while continually encouraging consumers to use mHealth technology to change health behaviors.

## Abbreviations

BCT: behavior change technique
HIIT: high-intensity interval training
mHealth: mobile health
MICT: moderate-intensity continuous training
PA: physical activity
RCT: randomized controlled trial
SM: mHealth self-monitoring

## Appendix

Multimedia Appendix 1 CONSORT-EHEALTH checklist (V 1.6.1).
